# Immuno-phenotyping of Canadian beef cattle: adaptation of the high immune response methodology for utilization in beef cattle

**DOI:** 10.1093/tas/txac006

**Published:** 2022-01-27

**Authors:** Nasrin Husseini, Shannon C Beard, Douglas C Hodgins, Christy Barnes, Elfleda Chik, Bonnie A Mallard

**Affiliations:** 1 Department of Pathobiology, University of Guelph, Guelph, Ontario N1G 2W1, Canada; 2 Bennett Equine Veterinary Services, Cambridge, Ontario, N1T 1T1, Canada; 3 Swine Health Professionals Ltd., Steinbach, Manitoba, R5G 1M1, Canada

**Keywords:** antibody-mediated immune response, beef cattle, cell-mediated immune response, health, high immune response, immune-competence

## Abstract

The high immune response (HIR) methodology measures the genetic performance of the adaptive immune system to identify and breed animals with balanced and robust immunity. The HIR methodology has previously been used in dairy and swine to reduce disease but has not been fully investigated in beef cattle. The first objective of the current study was to examine whether the HIR methodology as standardized for use in dairy cattle was appropriate for use in beef cattle. The second objective was to determine the earliest age for immune response phenotyping of beef calves. In this study, beef calves (*n* = 295) of various ages, as well as mature beef cows (*n* = 170) of mixed breeds, were immunized using test antigens to assess their antibody- (AMIR) and cell-mediated immune responses (CMIR). Heritability for AMIR and CMIR was estimated at 0.43 and 0.18, respectively. The HIR methodology was appropriate for use in beef cattle; beef calves as young as 2–3 wk of age were capable of mounting AMIR responses comparable with those seen historically in mature Holstein dairy cows. Three-week-old beef calves mounted CMIR responses comparable with those of Holstein cows, but 9-mo-old calves and mature beef cows had significantly higher CMIR responses than Holsteins. The HIR methodology can be used to measure both AMIR and CMIR in beef calves as young as 3 wk of age.

## INTRODUCTION

In the past, selection of livestock mainly focused on production traits with less attention on health traits. However, recently more attention is paid to animal health and welfare, particularly in light of consumer concern about food management systems and antibiotic treatment of livestock (Tirado et al., 2010). Vaccination and management strategies are used to improve animal health. Nonetheless, vaccine efficacy can still be a challenge for certain complex diseases, such as bovine respiratory disease (BRD; Anholt et al., 2017). Genetic selection is another approach to enhance livestock health. Studies have shown that selective breeding of dairy cattle for balanced, superior, and robust immune responses not only reduces the incidence of disease but also improves the quality of their milk and colostrum (Thompson-Crispi et al., 2014a; Fleming et al., 2016; Stear et al., 2017; Emam et al., 2019), as well as certain reproduction and growth traits (Mallard and Wilkie, 2007; Thompson-Crispi et al., 2012; Aleri et al., 2015). Additionally, Mallard and Wilkie (2007) have reported that high immune responder pigs reach market weight (100 kg) 10–12 d faster than low immune responders. Aleri et al. (2015) reported similar findings in their study of Australian Holstein heifers in which cattle with high immune responses (HIR) had higher daily weight gains compared with low immune response cattle.

The patented HIR (patent # US7258858B2) methodology measures the genetic performance of the adaptive immune system to identify and breed animals with balanced and robust immunity. Using HIR methodology, dairy cattle and pigs with superior immunity have been identified and bred for these heritable health traits (Mallard et al., 2015). This technology ranks animals by measuring the response of both arms of the adaptive immune system such as antibody- (AMIR) and cell-mediated immune responses (CMIR), and classifies individuals as high, average, or low responders. Thompson-Crispi et al. (2012, 2014a) reported that the incidence of disease in high immune responder dairy cattle is about half that of low immune responders. Additionally, these high responders are able to pass this fitness trait to future generations with a heritability similar to the trait of milk production (approximately 0.20–0.35, Thompson-Crispi et al., 2012; Larmer and Mallard, 2017). The HIR technology is utilized by the Semex Alliance under the tradename of Immunity+. Daughters of the HIR/Immunity+ dairy sires have lower incidence of disease with no adverse effects on production (Mallard et al., 2015; Larmer and Mallard, 2017).

Given the health benefits of selecting dairy cattle using the HIR methodology, the first objective of the current study was to determine if the standard HIR methodology (dose, time interval between sample collections, and protocol) developed for use in Holstein dairy cattle was appropriate for immune phenotyping of Canadian beef cattle of mixed breeds.

The second objective was to compare options as to the most practical age to phenotype beef cattle. In dairy cattle, the youngest age the HIR methodology is applied is 2 mo of age, based on the standardized dairy protocol. In the life cycle of beef cattle, there are limited opportunities to access cattle for phenotyping. There is a strong motivation to disperse cows with newborn calves to pastures or extensive range areas as early as possible to decrease spread of pathogens among the calves. Phenotyping is impractical under these pasture or range conditions. Phenotyping of young calves before movement to pasture might be a viable option. At the time of weaning (generally 6–7 mo of age), calves are subjected to stresses related to separation from the dam, adaptation to new feed sources, and deteriorating seasonal weather conditions and associated respiratory disease (in North America), which adversely affect immune responses. Administration of antigens during this time of maximum stress may fail to reflect the genetic ability of calves to respond immunologically (Richeson and Falkner, 2020). Thus the second objective of the current study was to compare phenotyping in very young calves (before dispersal to pastures or ranges), with phenotyping 9-mo-old calves that have completed the weaning process and become acclimated to post-weaning conditions.

It is expected that adoption of the HIR methodology, and breeding for inherited health traits in beef cattle will lower the incidence of important production diseases, such as BRD. The hypothesis of this study was that mature beef cattle of mixed breeds, as well as young calves, could be immune phenotyped for both AMIR and CMIR using HIR methodology.

## MATERIALS AND METHODS

All experimental procedures were approved by the Animal Care Committee of the University of Guelph under guidelines of the Canadian Council of Animal Care. All calves born at the Ontario Beef Research Centre (OBRC, operated by the University of Guelph) in 2016 and 2017 were enrolled in the current study. Cows and breeding heifers were vaccinated annually with five-way antiviral vaccines (bovine rhinotracheitis virus, bovine viral diarrhea virus types 1 and 2, parainfluenza virus type 3, and bovine respiratory syncytial virus) and bacterin containing five serotypes of *Leptospira*. Cows and breeding heifers were vaccinated annually 1 mo before the calving season with a vaccine containing inactivated bovine rotavirus, bovine coronavirus, K99 antigen positive *Escherichia coli*, and *Clostridium perfringens* type C. Calves were vaccinated in mid-September with a five-way antiviral vaccine (as described earlier), with a *Mannheimia haemolytica* toxoid, in addition to a multivalent *Clostridium chauvoei*, *Clostridium septicum*, *Clostridium novyi*, *Clostridium sordellii*, *Clostridium perfringens* types C and D, and *Histophilus somni* bacterin. Calves received booster vaccinations with these products 6 wk later, before weaning. The antigen preparation used in the HIR immune phenotyping protocol contains none of the antigens present in any of the vaccines received by the cows, breeding heifers or calves as listed earlier.

### Immuno-Phenotyping

Crossbred beef calves (Black Angus-, Red Angus-, Simmental-, and Piedmontese-crosses) born in April and May at the OBRC were enrolled in the current study. Artificial insemination was used extensively in this research herd. A total of 151 calves born in 2016 were sired by 44 different bulls, and 144 calves born in 2017 were sired by 36 different bulls. A total of 18 bulls were sires of one or more calves born in each of the study years. The proportion of Angus breeding in the calves varied from 0.125 to 0.875. Bull calves were castrated using rubber castration bands applied by 48 h after birth.

In 2016, calves were immuno-phenotyped starting at either 3 wk or 9 mo of age. Three weeks was chosen as the starting age for phenotyping of half of the calves in the first year of the study, because this was the oldest starting age compatible with completion of phenotyping before the anticipated date for movement of cows and calves to pasture in the spring. Immune phenotyping was performed in January for the second half of the calves when they were about 9 mo old, to avoid immune interactions with commercial vaccines, and to avoid phenotyping stressed or sick calves.

The dams of calves in this study were part of a concurrent nutritional study (five different treatment groups, plus one standard diet control group). Cows were maintained on low energy diets in winter and fed added supplements in the spring. Calves were allocated to be tested at 3 wk or 9 mo of age using systematic random allocation, as calves were born within each of the maternal nutritional groups. To avoid anamnestic responses, HIR methodology can be performed only once in the life of an animal.

Heifer and steer calves beginning at either 3 wk or 9 mo of age were immunized intramuscularly on day 0 to induce CMIR and AMIR according to the HIR methodology, using an antigen preparation containing both type 1 and type 2 antigens in adjuvant as described earlier (Thompson-Crispi et al., 2013). Blood samples were collected on days 0 and 14 for analysis of AMIR responses to type 2 antigen. Cutaneous delayed-type hypersensitivity (DTH) response was used to assess CMIR to type 1 test antigen. Double skin fold thickness (DSFT) of the left and right tail folds was measured on day 14 in triplicate with calipers (Harpenden skinfold calipers, Creative Health Products Inc., Ann Arbor, MI). An intradermal injection of 100 μL of saline solution was injected into the left skin fold and 100 μL of a solution of type 1 antigen was injected into the right skin fold. After 24 h, DSFT was measured in triplicate for skin of both left and right tail folds. Skin fold measurements were entered into statistical models as described by Thompson-Crispi et al. (2012). The log_10_ (DSFT at 24 h divided by DSFT at 0 h) for the antigen-injected site was the outcome of interest. The corresponding log_10_ ratio for the saline-injected site was entered into models as a covariate.

An enzyme-linked immunosorbent assay (ELISA) was used to measure AMIR in sera to the type 2 test antigen. The positive control for the ELISA was pooled sera from previously immunized cows and the negative control was fetal bovine serum. Sera were assayed in quadruplicate. Alkaline phosphatase conjugated monoclonal antibodies to bovine IgG (clone BG18, Sigma-Aldrich, Oakville, Ontario, Canada, Catalog #A7554) were used to detect serum IgG antibodies bound to type 2 antigen. Alkaline phosphatase substrate (*p*-nitrophenyl phosphate, Sigma-Aldrich, catalog #N1891-50SET) was used to detect bound conjugate. ELISA plates were read at wavelengths of 405 and 630 nm when the mean optical density (OD) of the positive control wells was near 1.000. OD at 630 nm were subtracted from OD at 405 nm. The mean OD of negative control wells was then subtracted from the OD of test wells. ODs of test sera were standardized to the positive control by dividing the mean OD of the positive wells. Adjusted OD for day 0 sera (collected before administration of type 2 antigen) had a mean of 0.023 ± (standard deviation) 0.024. The same protocol was followed for all sera reported in the present study.

In 2017, crossbred beef calves were allocated to be immuno-phenotyped at either 1 wk, 3 wk, or 9 mo of age using systematic random allocation as calves were born. The objective of this protocol was to evaluate whether calves could be tested using the established HIR methodology, at less than 3 wk of age, comparing their AMIR and CMIR to those of 9-mo-old calves.

When two-thirds of the cows had calved, it became clear that calves phenotyped in the first week of life had significantly lower antibody responses to type 2 antigen, compared with 3-wk-old calves. As a result, calves born subsequently, which would have been allocated for phenotyping at 1 wk of age were phenotyped instead at 2 wk of age in order to better define the lower age limit for antibody responses. Final allocation of calves was 32 calves phenotyped at 1 wk of age (2–7 d, mean 4.0 ± 1.8 d), 16 calves at 2 wk of age (11–16 d, mean 13 ± 2.0 d), 49 calves at 3 wk of age (21–26 d, mean 22.6 ± 1.4 d), and 47 calves at 9 mo of age (mean 8.5 ± 0.5 mo).

In both years, 2016 and 2017, some cow–calf pairs were on pasture over the summer and some remained in open-shed housing. These effects were evaluated in the statistical model as described later.

Additionally, in order to immuno-phenotype fully mature cows as another point of comparison with younger animals, 170 mixed breed (Black Angus-, Red Angus-, Simmental-, and Piedmontese-crosses) mature beef cows were tested, 126 of them being the dams of the calves born in 2017. The proportion of Angus breeding in the cows varied from 0.063 to 0.938. Cows were tested as described earlier from October to November 2017, 5–6 mo after their calves were tested using the HIR methodology.

Finally, historical immune response data on mature Canadian Holstein cows (*n* = 3,304) from 71 herds across Canada, phenotyped as an extension of a previous study (Thompson-Crispi et al., 2014b), using the same HIR methodology (with the modification that serum antibody responses on day 14 were used to evaluate AMIR responses) were used to compare AMIR and CMIR among age groups and between breeds.

### General Linear Models

A SAS (SAS 9.4, SAS Institute Inc., Cary, NC, USA) general linear model (GLM) was used to examine fixed effects influencing AMIR and CMIR of beef cattle of varying ages as follows:


$$\begin{array}{ll} y_{ijklmnopq}= & \;\mu +\beta \times cont_{i}+agecl_{j}+s_{k}+ang_{l}+ra_{m}+si_{n}\\ & +pi_{o}+n_{p}+hou_{q}+e_{ijklmnopq}, \end{array}$$

where $y_{ijklmnopq}$ is the AMIR (log day 14 antibody response OD) or CMIR (log 24 h change in skin fold thickness at the test site); μ is the overall mean; β is the regression coefficient; $cont_{i}$ is the control for AMIR (log day 0 antibody response OD) or CMIR (log 24 h change in skin fold thickness at the control site) fitted as a covariate; *agecl*_*j*_ is the fixed effect of age class (where j = mature beef cows, 9-mo-old calves, 3-wk-old calves, 2-wk-old calves, or 1-wk-old calves); $s_{k}$ is the fixed effect of sex (where k= male or female); $ang_{l}$ is the fixed effect of Angus (where l*=* number of Black Angus out of 32 possible progenitors; data from bioTrack, http://agsights.com/what-is-go360-biotrack/, used to generate the progenitors going back five generations); $ra_{m}$ is the fixed effect of Red Angus (where m = number of Red Angus out of 32 possible progenitors); $si_{n}$ is the fixed effect of Simmental (where n = number of Simmental out of 32 possible progenitors); $pi_{o}$ is the fixed effect of Piedmontese (where o = number of Piedmontese out of 32 possible progenitors); $n_{p}$ is the fixed effect of nutrition (where p denotes nutrition groups from 1 to 6 depending on the diet); $hou_{q}$ is the fixed effect of summer housing (where q = open shed or pasture); and $e_{ijklmnopq}$ is the residual error.

Variables with *P* > 0.05 were removed from the model. Results were considered to be statistically significant if *P* ≤ 0.05. Interactions were tested and remained in the model if *P* < 0.05. Least-squares means (LSmeans) were used to indicate the different AMIR and CMIR responses of beef calves, and mature beef cows.

Subsequently, another SAS (SAS/STAT, 1999) GLM was used to examine fixed effects influencing AMIR and CMIR of beef cattle of varying ages with Holstein cow data added to the model as follows:


$$\begin{array}{ll} y_{ijklmnopq}= & \;\mu +\beta \times cont_{i}+agecl_{j}+s_{k}+ang_{l}+ra_{m}+si_{n}\\ & +pi_{o}+n_{p}+hou_{q}+e_{ijklmnopq}, \end{array}$$

where the variables are as described earlier except where $agecl_{j}$ is the fixed effect of age class (where j = mature beef cows, 9-mo-old beef calves, 3-wk-old beef calves, 2-wk-old calves, 1-wk-old calves, or mature Holstein cows).

PROC Univariate (SAS) was used to check the normality of all data sets (OD and the DSFT measurements were log_10_ transformed).

### Heritability Analysis

Heritability estimates for AMIR and CMIR were calculated using data from 3-wk-old calves, 9-mo-old calves, and mature beef cows (*n* = 417). The model used for AMIR was


$$\begin{array}{ll} y_{ijklm}= & \;\mu +\beta \times cont_{i}+agecl_{j}+s_{k}\\ & +ang_{l}+agecl\;*\;ang\;+a_{m}+e_{ijklm}, \end{array}$$

where $y_{ijklm}$ is the log day 14 antibody response OD, μ is the overall mean, β is the regression coefficient, $cont_{i}$ is the control (log day 0 antibody response OD) fitted as a covariate, $agecl_{j}$ is the fixed effect of age class (where j = mature cows, 9-mo-old calves, or 3-wk-old calves), $s_{k}$ is the fixed effect of sex (where k = male or female), $ang_{l}$ is the fixed effect of Angus breed proportion (where l = low [0–9 progenitors], medium [10–19 progenitors], high [20–29 progenitors]), $a_{m}$ is the random effect of animal, and $e_{ijklm}$ is the residual error.

The model used to calculate heritability estimates for CMIR was


$$\begin{array}{l} y_{ijm}=\mu +\beta \times cont_{i}+agecl_{j}+a_{m}+e_{ijm}, \end{array}$$

where $y_{ijk}$ is the log 24 h change in skin fold thickness at the test site, μ is the overall mean, β is the regression coefficient, $cont_{i}$ is the control (log 24 h change in skin fold thickness at the control site) fitted as a covariate, $agecl_{j}$ is the fixed effect of age class (where j = mature cows, 9-mo-old calves, or 3-wk-old calves), $a_{m}$ is the random effect of animal, and $e_{ijm}$ is the residual error.

In matrix form, the single trait animal model for both AMIR and CMIR was


$$\begin{array}{l} y=Xb+Za+e, \end{array}$$

where **y** is the vector of observations for AMIR or CMIR, **X** is the incidence matrix relating observations to fixed effects, **b** is the vector of fixed effects, **Z** is the incidence matrix relating observations to random effects, **a** is the vector of random additive genetic effects of animal, and **e** is the vector of random residual effects. The expectations and assumed variances are **E**(**y**) = **Xb**, **E**(**a**) = **E**(**e**) = 0, **V**(**a**) = **G, V**(**e**) = **R,** cov(**a, e**’) = 0, and **V**(**y**) **= ZGZ’** + **R,** where **R** is the direct product between an identity matrix of order of the number of observations and the matrix of error variances and covariances (**I** ⊗ **R**_**0**_), and **G** is the direct product between the additive relationship matrix (**A**) constructed from the pedigree of the animals and their eight generation ancestors (*n* = 1,350) and the matrix of genetic variances and covariances (**A** ⊗ **G**_**0**_).

Heritability estimates were calculated as


$$\begin{array}{l} h^{2}={{}_{\sigma a}}^{2}/({\sigma_{a}}^{2}+{\sigma_{e}}^{2}), \end{array}$$

using univariate linear animal models in ASReml 4.1 (Gilmour et al., 2015).

## RESULTS

### GLM Analyses: Comparing AMIR and CMIR of Beef Cattle of Various Ages

**Figure 1.**
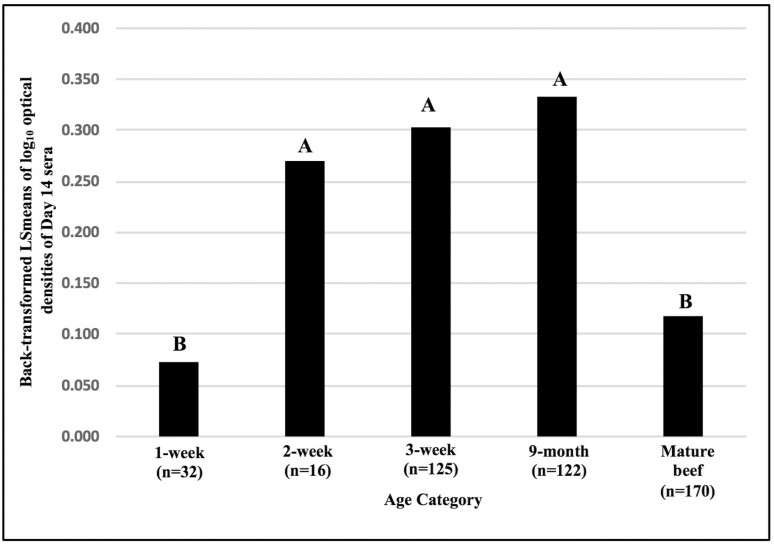
AMIR, based on ELISA OD of day 14 sera, in 1-, 2-, and 3-wk-old, and 9-mo-old mixed beef breed calves born in 2016 and 2017 with mature beef cows following immunization with an HIR type 2 antigen. Day 14 log_10_ transformed OD were analyzed using GLM. log_10_ OD for day 0 sera were entered in statistical models as covariates. LSmeans were back-transformed to original units (OD) for graphing purposes. Columns with the same letter do not differ significantly; columns with different letters differ significantly at *P* < 0.05.

The model for AMIR was significant (Table 1, *P* < 0.0001) and accounted for 16% of the total variation in this trait (*R*^2^ = 0.16). Comparison of LSmeans of log_10_ transformed day 14 antibody responses of calves tested at 1, 2, 3 wk, and 9 mo of age with those of mature beef cows of mixed breeds, indicated that 1-wk-old calves had the lowest AMIR responses. Specifically, the AMIR responses at 1 wk of age were significantly lower than those of 2-wk-, 3-wk-, and 9-mo-old-calves (Figure 1). Comparison of antibody concentrations in sera of 1-wk-old calves between day 0 (geometric mean OD = 0.010, 95% confidence interval [CI] = (0.006, 0.016)) and day 14 (geometric mean OD = 0.066, CI = (0.037, 0.118)), suggests that calves of this age can mount antibody responses to the type 2 antigen, but responses are low in magnitude. The AMIR of 2-wk-, 3-wk-, and 9-mo-old-calves were significantly higher than those of mature beef cows. However, there was not a significant difference between responses of 1-wk-old calves and those of mature beef cows even though the 1-wk-old calves had the lowest geometric mean antibody responses. Comparison of AMIR in sera of mature beef cows between day 0 (geometric mean OD = 0.021, CI = (0.019, 0.024)) and day 14 (geometric mean OD = 0.148, CI = (0.118, 0.187)) suggests that mature beef cows can mount antibody responses to the type 2 antigen, but responses are relatively low in magnitude.

**Table 1.** GLM for AMIR on day 14, in all age groups (1-, 2-, and 3-wk-old, and 9-mo-old calves with mature beef cows) by number of Angus progenitors, out of 32 possible progenitors (data from bioTrack^∗^ for the progenitors going back five generations, Angus category being either low [0–9 progenitors], medium [10–19 progenitors], or high [20–32 progenitors])

Variable	Model	log_10_ AMIR day 0	Age	Angus category	Angus category∗age	*R* ^2^
log_10_ AMIR day 14	*P* < 0.0001	*P* < 0.0001	*P* < 0.0001	*P* = 0.55	*P* = 0.01	16%

BioTrack is designed to collect and help analyze different types of data generated on farm (http://agsights.com/what-is-go360-biotrack/)

**Figure 2.**
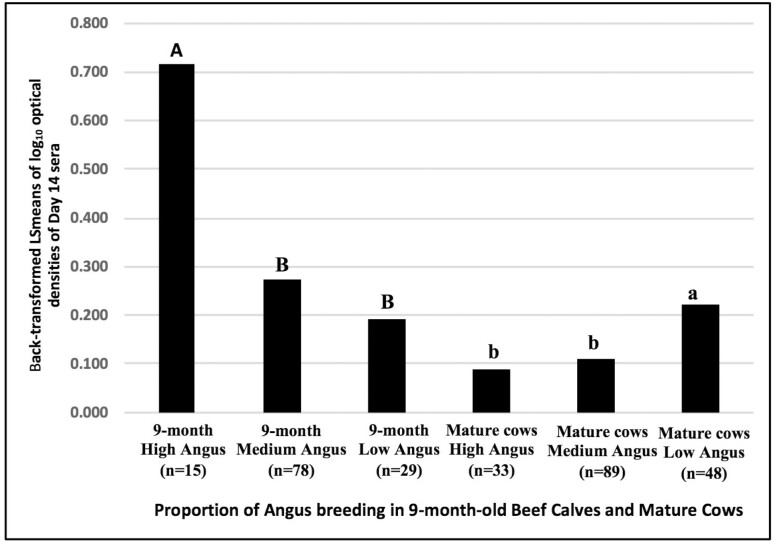
AMIR, based on ELISA OD of day 14 in sera, in 9-mo-old mixed breed beef calves (born in 2016 and 2017) and mature beef cows classified by the number of Angus progenitors, out of 32 possible progenitors (at the fifth generation). Classifications: high Angus (20–32 Angus progenitors), medium Angus (10–19 Angus progenitors), low Angus (0–9 Angus progenitors). Data are derived from herd records maintained in AgSights Go360 bioTrack livestock management software (http://agsights.com/what-is-go360-biotrack/). Day 14 log_10_ transformed OD were analyzed using GLM. log_10_ OD for day 0 sera were entered in statistical models as covariates. LSmeans were back-transformed to original units (OD) for graphing purposes. Capital letters indicate comparisons among 9-mo-old beef calves; lower case letters indicate comparisons among mature beef cows. Within each group, columns with the same letter do not differ significantly; columns with different letters differ significantly at *P* < 0.05.

After observing a significant interaction between the age of testing and proportion of Angus (Table 1, *P* = 0.01, number of Black Angus progenitors, out of 32 possible progenitors [data from bioTrack] for the progenitors going back five generations, with low [0–9 progenitors], medium [10–19 progenitors], and high [20–29 progenitors]), LSmeans for the interaction were examined within each age category separately (1-, 2-, and 3-wk-old, 9-mo-old, and mature beef cows). No effect of proportion of Angus was observed in 1-, 2-, and 3-wk-old calves. However, 9-mo-old calves with the high proportion of Angus had significantly higher AMIR response compared with those with medium (*P* = 0.01) and low (*P* = 0.003). In contrast, mature cows with low proportion of Angus had significantly higher AMIR response compared with high (*P* = 0.005) and medium proportions (*P* = 0.01, Figure 2). There was no interaction of age and proportion for other breeds in the model.

**Figure 3.**
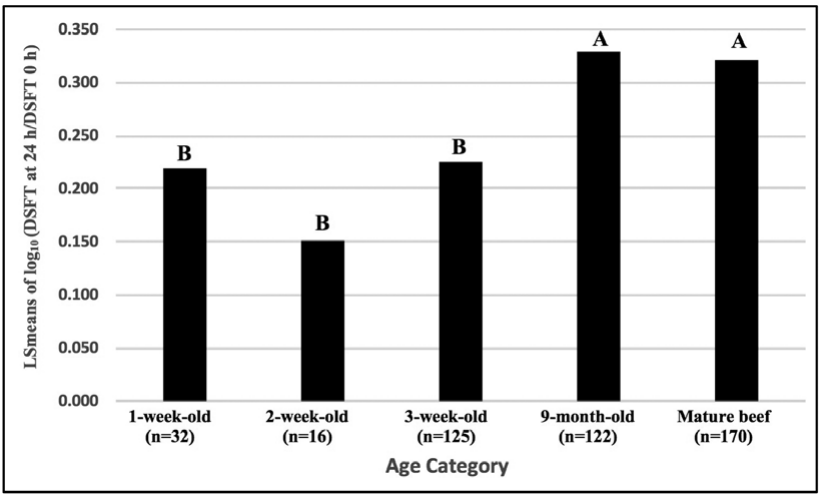
Comparison of CMIR based on DTH responses to type 1 test antigen in 1-, 2-, and 3-wk-old, and 9-mo-old mixed breed beef calves (born in 2016 and 2017), and mature beef cows, 14 d after immunization with an HIR type 1 antigen. Antigen was injected intradermally into the skin of one tail fold on day 14, and saline control solution into the contralateral fold. Changes in DSFT at antigen and control sites after 24 h were analyzed using GLM. The log_10_ (DSFT at 24 h divided by DSFT at 0 h) for the antigen-injected site was the outcome of interest. The corresponding log_10_ ratio for the saline-injected site was entered into models as a covariate. Columns with the same letter do not differ significantly; columns with different letters differ significantly at *P* < 0.05. LSmeans = least square means.

**Figure 4.**
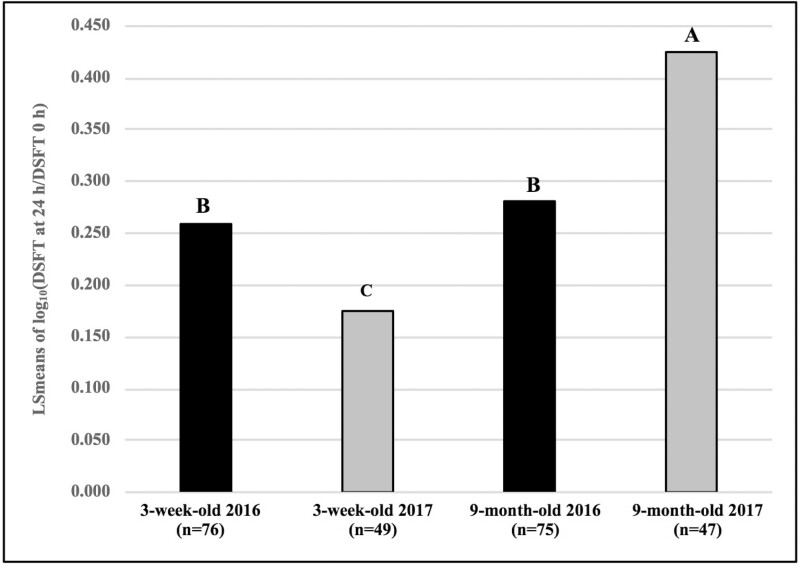
Comparison of CMIR based on DTH responses to type 1 test antigen in 3-wk-old, and 9-mo-old mixed breed beef calves (born in 2016 and 2017), 14 d after immunization with an HIR type 1 antigen. Antigen was injected intradermally into the skin of one tail fold on day 14, and saline control solution into the contralateral fold. Changes in DSFT at antigen and control sites after 24 h were analyzed using GLM. The log_10_ (DSFT at 24 h divided by DSFT at 0 h) for the antigen-injected site was the outcome of interest. The corresponding log_10_ ratio for the saline-injected site was entered into models as a covariate. Columns with the same letter do not differ significantly; columns with different letters differ significantly at *P* < 0.05. LSmean = least square mean.

The model for CMIR was significant (Table 2, *P* < 0.0001) and accounted for (*R*^2^ = 0.18) 18% of the total variation in this trait. Age was significant in the CMIR analysis (*P* < 0.0001). LSmeans of CMIR results indicated that 1-, 2-, and 3-wk-old calves had similar CMIR, however the responses were significantly lower than those of 9-mo-old and mature beef cows (Figure 3). It was of interest whether CMIR responses were similar among calves born in 2016 and 2017. Responses of 3-wk-old and 9-mo-old calves in the 2 yr were compared (Table 2). There was a significant interaction between age and birth year (age category∗birthyear). Among calves born in 2016, CMIR in calves tested at 3 wk and 9 mo of age were similar, but among calves born in 2017, calves tested at 9 mo of age had significantly higher CMIR than those tested at 3 wk of age (Figure 4).

**Table 2.** GLM for CMIR on day 15

a) CMIR in 1-, 2-, and 3-wk-old, and 9-mo-old calves and mature beef cows				
Variable	Model	log_10_ DSFT ratio control site^∗^	Age category	*R* ^2^
log_10_ DSFT ratio antigen site^†^	*P* < 0.0001	*P* < 0.0001	*P* < 0.0001	18%

b) CMIR in 3-wk-old, and 9-mo-old calves: 2016 vs. 2017				
Variable	Model	log_10_ DSFT ratio control site	Age category∗year	*R* ^2^
log_10_ DSFT ratio antigen site	*P* < 0.0001	*P* < 0.001	*P* < 0.0001	35%

For the control site: log_10_ (DSFT at 24 h/DSFT at 0 h) was used as a covariate.

For the antigen site: log_10_ (DSFT at 24 h/DSFT at 0 h) was the outcome of interest.

### Comparing AMIR and CMIR of Beef Cattle of Various Ages with Holstein Cows

**Figure 5.**
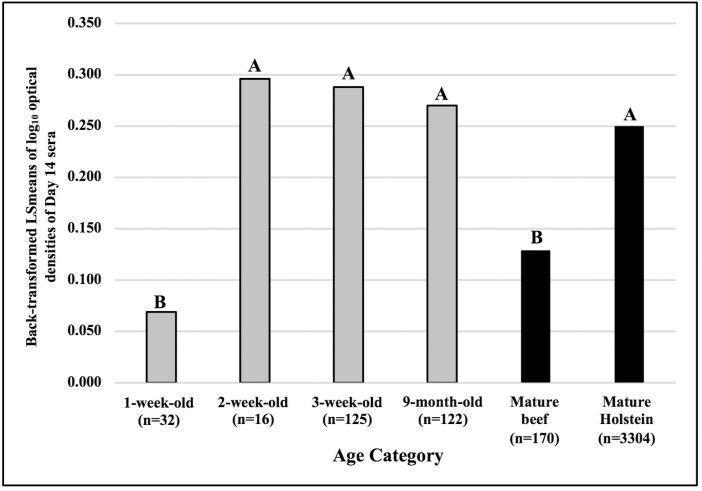
AMIR, based on ELISA OD of day 14 sera, of 1-, 2-, and 3-wk-old, and 9-mo-old mixed breed beef calves (born in 2016 and 2017) with mature beef and Holstein cows on day 14 following immunization with an HIR type 2 antigen. Day 14 log_10_ transformed OD were analyzed using GLM. log_10_ OD for day 0 sera were entered in statistical models as covariates. LSmeans were back-transformed to original units (OD) for graphing purposes. Columns with the same letter do not differ significantly; columns with different letters differ significantly at *P* < 0.05.

The model for AMIR was significant (Table 3, *P* < 0.0001) and accounted for (*R*^2^ = 0.09) 9% of the total variation in this trait. Calves tested at 1-wk of age had significantly lower AMIR responses than 2-wk-, 3-wk-, and 9-mo-old-calves, as well as mature beef cows and Holstein dairy cows (Figure 5). The AMIR of 2-wk-, 3-wk-, and 9-mo-old-calves were similar to those of Holstein cows. Mature beef cows had significantly lower AMIR than Holsteins. Overall, AMIR of 1-wk-old calves were low (Figure 5), and it is recommended to test AMIR after calves are at least 2 wk of age.

**Table 3.** GLM for AMIR on day 14, for age effect (1-, 2-, and 3-wk-old, and 9-mo-old calves with mature beef cows and mature Holstein cows)

Variable	Model	log_10_ AMIR day 0	*R* ^2^
log_10_ AMIR day 14	*P* < 0.0001	*P* < 0.0001	9%

**Figure 6.**
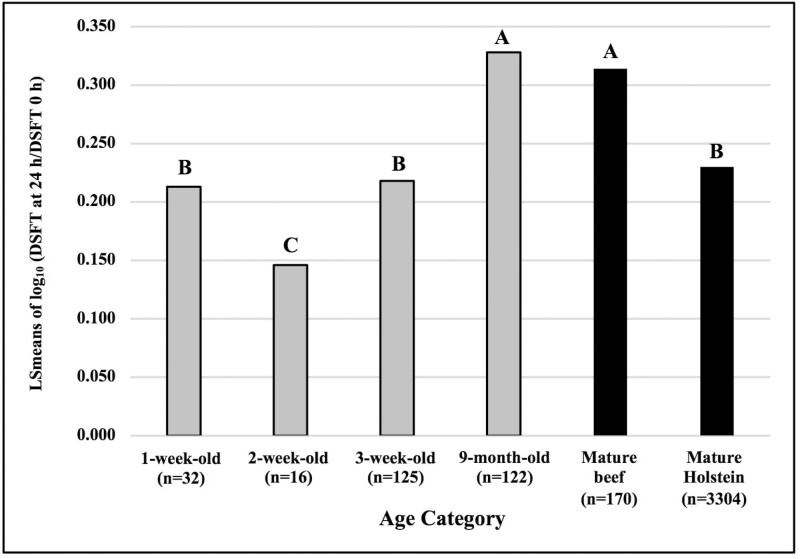
Comparison of CMIR based on DTH responses to type 1 test antigen in 1-, 2-, and 3-wk-old, and 9-mo-old mixed breed beef calves (born in 2016 and 2017), with mature beef and Holstein cows, 14 d after immunization with an HIR type 1 antigen. Antigen was injected intradermally into the skin of one tail fold on day 14, and saline control solution into the contralateral fold. Changes in DSFT at antigen and control sites after 24 h were analyzed using GLM. The log_10_ (DSFT at 24 h divided by DSFT at 0 h) for the antigen-injected site was the outcome of interest. The corresponding log_10_ ratio for the saline-injected site was entered into models as a covariate. Columns with the same letter do not differ significantly; columns with different letters differ significantly at *P* < 0.05. LSmean = least square mean.

The model for CMIR was significant (Table 4, *P* < 0.0001) and accounted for 9% of the total variation in this trait (*R*^2^ = 0.09). Testing age was significant (*P* < 0.0001). LSmeans of CMIR results indicated that 1-wk- and 3-wk-old calves had CMIR similar to those of mature Holstein cows (Figure 6). Two-wk-old calves had significantly lower CMIR compared with 1-wk- and 3-wk-old calves. Nine-mo-old calves and mature beef cows had significantly higher CMIR than historic data from mature Holstein cows.

**Table 4.** GLM for CMIR on day 15, for age effect (1-, 2-, 3-wk-old, and 9-mo-old calves with mature beef cows and mature Holstein cows)

Variable	Model	log_10_ DSFT ratio control site^∗^	Age	*R* ^2^
log_10_ DSFT ratio antigen site^†^	*P* < 0.0001	*P* < 0.0001	*P* < 0.0001	9%

For the control site: log_10_ (DSFT at 24 h/DSFT at 0 h) was used as a covariate.

For the antigen site: log_10_ (DSFT at 24 h/DSFT at 0 h) was the outcome of interest.

In summary, in comparison to historic data from over 3,300 mature Holstein cows phenotyped across Canada, 3-wk-old calves had AMIR and CMIR comparable with those of mature Holsteins; 9-mo-old beef calves had AMIR comparable with that of Holsteins but significantly higher CMIR than that of Holsteins.

### Heritability Estimates

Heritabilities for AMIR and CMIR were estimated at 0.43 (SEM = 0.10) and 0.18 (SEM = 0.10), respectively. Although these estimates are from a relatively small sample size, they are consistent with those previously found for Holstein dairy cattle and Angus beef cattle.

## DISCUSSION

Various approaches have been taken to reduce livestock disease incidence, some of them focusing on improving health using genetics, facilitating permanent improvements that can be passed on to future generations. The HIR methodology utilized in both research and commercial dairy herds permits selection of individuals with robust and balanced immune responses in order to improve health and welfare of animals (Mallard et al., 2015; Larmer and Mallard, 2017). Although the HIR methodology has previously been applied in dairy, this test method has not been fully validated for beef cattle. Therefore, the first objective of this study was to determine if the standard HIR methodology developed for use in Holstein dairy cattle was appropriate for use in beef cattle of mixed breeds. Historically, the standard HIR methodology was optimized to induce both AMIR and CMIR of sufficient magnitude and with sufficient variation to permit classification of superior and inferior responses. A historic dataset of AMIR and CMIR responses of over 3,300 mature Holstein cows provided a frame of reference for the current study as to what constitute adequate responses. In the current work, 2-wk- and 3-wk-old and 9-mo-old beef calves generated AMIR responses comparable with those of mature Holstein cows. Three-wk-old calves had CMIR comparable with mature Holstein cows, and 9-mo-old calves and mature beef cows had CMIR significantly higher than those of Holsteins. These findings confirm that the HIR methodology optimized for use in Holsteins is indeed appropriate to induce AMIR and CMIR in beef cattle if applied to beef cattle of sufficient age.

In dairy, the youngest age the HIR methodology applied is 2 mo of age, based on the standardized dairy protocol. The second objective of this study was to determine the youngest age at which beef calves could be immuno-phenotyped. In North America, dairy cattle are readily accessible for phenotyping activities throughout the year since cows are bred and milked without regard to the seasons, and housing and restraint facilities are not factors limiting accessibility. However, in traditional beef management systems in North America, beef calves are born in springtime and are maintained on pasture or range land during the summer making access to cattle for phenotyping purposes difficult. Consequently, the goal of finding the youngest age that beef calves could be immuno-phenotyped prior to their departure to pasture or range was a relevant one.

Fetal calves have all the components of the adaptive immune system (Wilson et al., 1996). However, their immune system is still naïve; the number of immune cells is low as well as their functionality (Wilson et al., 1996; Simon et al., 2015). Although lymphocytes from fetal calves can respond to stimulation with mitogens by 188–253 d gestation (Wilson et al., 1996), yet the immune system does not appear to be fully functional until 2–4 wks after birth to make a mature-like immune response (Tierney and Simpson-Morgan, 1997). During the neonatal period (0–10 d after birth) lymphocytes and monocytes are substantially lower in numbers (3.5 × 10^9^/L and 0.4 × 10^9^/L, respectively) than in mature cows (7.8 × 10^9^/L and 0.8 × 10^9^/L; Knowles et al., 2000). Neonatal calves also have approximately 30% lower B cell counts, and only by 20 d after birth do counts reach adult levels (Barrington and Parish, 2001). Cytokines and other immuno-regulatory factors such as microRNAs, transferred with colostrum to neonates may also modulate the immune system (Okada et al., 2010; Hodgins and Shewen, 2012; Emam et al., 2019).

Following birth, calves depend on the passive immunity they receive from their dams to activate and regulate their immune responses, as well as to passively fight infection, but the presence of antigen-specific antibodies can suppress active antibody responses of neonatal calves (Chase et al., 2008). In the current study, 1-wk-old beef calves had significantly lower AMIR responses to type 2 antigen, than calves of 2 wk of age and older. The type 2 antigen utilized in the HIR has been chosen so that environmental or immunization exposure of cattle to this antigen is unlikely. Sera collected from calves phenotyped in the first week of life had a day 0 AMIR geometric mean of 0.010, consistent with negligible transfer of maternal IgG specific for the type 2 antigen. Thus the low AMIR responses by day 14 in these calves should not be attributed to the effects of maternal antibodies. It is possible that the kinetics of AMIR in these youngest calves may have been slower than in older cattle (and that peak titers might occur after day 14), but serum samples are not available to test this hypothesis.

In late gestation and near to birth, concentrations of corticosteroids increase in the bovine fetus to initiate parturition. Because of higher concentrations of cortisol in neonates, near birth and after, phagocytic activity and other immune functions remain low for up to 10 d after birth (Barrington and Parish, 2001; Hodgins and Shewen, 2012). Plasma cortisol concentrations reach a maximum concentration (193–331 nmol/L) at birth and return to basal levels approximately 10 d after birth. Mean cortisol concentrations of less than 20 nmol/L in plasma have been reported for tame beef bulls and heifers between 7 and 12 mo of age (Henricks et al., 1984; Hickey et al., 2003) and concentrations of 14–28 nmol/L have been reported for lactating cows (Hodgins and Shewen, 2012). Immune phenotyping of beef cattle during periods of high stress such as at branding, dehorning, castration, and/or weaning is contraindicated due to the higher concentrations of corticosteroids associated with these stresses, and their recognized immune suppressive effects (Richeson and Falkner, 2020).

At the outset of this study, 3 wk was chosen for practical reasons as the age for testing young beef calves to help ensure calves would not be on pasture or range land in a commercial herd. The age at which calves can respond to a particular antigen will vary with the antigen, the dose, and the adjuvant (Kirkpatrick et al., 2008). Therefore, immune responses were evaluated in the context of the established HIR methodology. Immuno-phenotyping results indicated that calves tested at 3 wk were able to mount AMIR similar to those of 9-mo-old calves and Holstein cows, and significantly higher than those of mature beef cows.

Calves immuno-phenotyped at 3 wk of age had CMIR responses comparable with those of mature Holstein cows (Figure 6). The large historic Holstein dataset (over 3,300 cows) can be considered a point of reference for the magnitude of CMIR responses that are acceptable for analysis of the genetic component of CMIR using the HIR methodology. In contrast, 9-mo-old beef calves and mature beef cows had significantly higher CMIR responses than Holstein cows. Mature beef cattle in commercial testing for Immunity+ have also been noted to have higher CMIR than dairy (Semex Alliance, Personal Communication). These findings underscore the importance of immuno-phenotyping groups of cattle of consistent age that have been raised in a common environment, to permit quantitation of the genetic components of observed variation in immune responsiveness.

Differences in CMIR phenotypes may be due to various environmental effects, including nutrition. The diet of suckling calves certainly differs from the rations fed to weaned calves in feedlots. In the current study, in the university beef herd, there was a nutritional study in progress in the dams of calves born in 2016 and 2017, with the nutrition that their dams received being different in these years. However, modeling indicated that the effects of nutrition were not significant (data not shown). Nonetheless other studies have reported dietary effects on immune response, especially cell-mediated immunity (Marcos et al., 2003).

Results also indicated a significant interaction between age category and proportion of Angus. Nine-mo-old calves with high proportion of Angus had significantly higher AMIR compared with those with medium and low. In contrast, mature cows with low proportion of Angus had significantly higher AMIR compared with those with medium and high. Engle et al. (1998) examined the effects of breed on immune responses of Angus and Simmental calves inoculated with infectious bovine rhinotracheitis virus via the intranasal route and reported that production of cytokines and fever was higher in Angus calves than in Simmental calves. They also observed a breed∗time interaction in antibody production following antigenic challenge of these calves (Angus and Simmental) with IgG titers in Angus calves peaking 7 d after injection compared with 14 d for Simmental. Together these results indicate that differences in immune responses among beef breeds are not unprecedented.

In the current study, heritability estimates for AMIR and CMIR were 0.43 (SEM = 0.10) and 0.18 (SEM = 0.10), respectively. Although these estimates are from a relatively small sample size, they are consistent with those previously found for dairy and beef cattle. For example, heritability estimates for AMIR and CMIR calculated from 445 Holsteins across Canada ranged from 0.16 to 0.41 and for CMIR was estimated at 0.19 (Thompson-Crispi et al., 2012). Other estimates of heritability for AMIR and CMIR in Holsteins range from 0.32 to 0.64 and 0.19 to 0.49, respectively, in line with the estimates calculated in this study (Wagter et al., 2000; Hernández et al., 2006). Heritability calculations for AMIR and CMIR in 1,100 Australian Angus calves were estimated at 0.32 and 0.27, respectively (Hine et al., 2019). The heritability estimations in this study suggest that immune response in beef cattle is moderately heritable and indicate its potential for genetic selection as has been found in Holstein dairy cattle (Larmer and Mallard, 2017).

## CONCLUSION

New and novel approaches that do not rely on antibiotics are essential to improve animal health and wellbeing. The HIR methodology has been shown effective for immuno-phenotyping of dairy and swine. This method can be used to classify individual animals based on their ability to make AMIR and CMIR, with those having the highest adaptive immune responses having the lowest occurrence of infectious disease. However, the HIR methodology had not previously been evaluated in beef cattle of various ages. The first objective of the current study was to examine whether the HIR methodology as standardized for use in dairy cattle was appropriate for use in beef cattle. The second objective was to determine the earliest age at which HIR methodology could be used to assess immune function of beef calves.

The results indicate that the HIR methodology established for Holstein dairy cattle and used successfully for years, can also be applied in beef cattle. Immuno-phenotyping for AMIR can be performed in beef calves as young as 2 wk of age and immuno-phenotyping for CMIR as young as 3 wk. Three weeks of age is therefore the youngest age recommended to evaluate both AMIR and CMIR. It was noted that mature beef cows have significantly lower AMIR than beef calves and mature Holstein cows, and mature beef cows and 9-mo-old beef calves both had significantly higher CMIR than historically encountered in Holstein cows. These findings should motivate more detailed, longitudinal studies in purebred beef cattle to examine how AMIR and CMIR drift and diverge as beef cattle age. Clearly, to analyze the genetic component of immune responses, it is critical to evaluate AMIR and CMIR within subpopulations of beef cattle of consistent age that have been raised within the same environment.

Heritability estimates for AMIR and CMIR were 0.43 (SEM = 0.10) and 0.18 (SEM = 0.10), respectively, and were consistent with estimates previously found for dairy and beef cattle suggesting that genetic improvement of immune responsiveness is probable for beef cattle.

## References

[CIT0001] Aleri, J. W., B. C. Hine, M. F. Pyman, P. D. Mansell, W. J. Wales, B. M. Mallard, and A. D. Fisher. 2015. Assessing adaptive immune response phenotypes in Australian Holstein-Friesian heifers in a pasture-based production system. J. Anim. Sci. 93:3713–3721. doi:10.2527/jas2015-9078 26440037

[CIT0002] Anholt, R. M., C. Klima, N. Allan, H. Matheson-Bird, C. Schatz, P. Ajitkumar, S. J. Otto, D. Peters, K. Schmid, M. Olson, et al. 2017. Antimicrobial susceptibility of bacteria that cause bovine respiratory disease complex in Alberta, Canada. Front. Vet. Sci. 4:1–10. doi:10.3389/fvets.2017.00207 29255716 PMC5723070

[CIT0003] Barrington, G. M., and S. M. Parish. 2001. Bovine neonatal immunology. Vet. Clin. North Am. Food Anim. Pract. 17:463–476. doi:10.1016/s0749-0720(15)30001-3 11692503 PMC7135619

[CIT0004] Chase, C. C., D. J. Hurley, and A. J. Reber. 2008. Neonatal immune development in the calf and its impact on vaccine response. Vet. Clin. North Am. Food Anim. Pract. 24:87–104. doi:10.1016/j.cvfa.2007.11.001 18299033 PMC7127081

[CIT0005] Emam, M., A. Livernois, M. Paibomesai, H. Atalla, and B. A. Mallard. 2019. Genetic and epigenetic regulation of immune response and resistance to infectious diseases in domestic ruminants. Vet. Clin. Food Anim. 35:405–429. doi:10.1016/j.cvfa.2019.07.002 31590895

[CIT0006] Engle, T. E., J. W. Spears, T. T. Brown, and K. E. Lloyd. 1998. Effect of breed (Angus vs Simmental) on immune function and response to a disease challenge in stressed steers and preweaned calves. J. Anim. Sci. 77(3):516–521. doi:10.2527/1999.773516x 10229346

[CIT0007] Fleming, K., D. C. Hodgins, F. Miglior, M. Corredig, and B. A. Mallard. 2016. Short communication: variation of total immunoglobulin G and beta-lactoglobulin concentrations in colostrum and milk from Canadian Holsteins classified as high, average, or low immune responders. J. Dairy Sci. 99:2358–2363. doi:10.3168/jds.2015-9707 26774725

[CIT0008] Gilmour, A. R., B. J. Gogel, B. R. Cullis, S. J. Welham, and R. Thompson. 2015. ASReml user guide release 4.1. Hemel Hempstead (UK): VSN International Ltd.

[CIT0009] Henricks, D. M., J. W. Cooper, J. C. Spitzer, and L. W. Grimes. 1984. Sex differences in plasma cortisol and growth in the bovine. J. Anim. Sci. 59(2):376–383. doi:10.2527/jas1984.592376x 6480533

[CIT0010] Hernández, A., V. Quinto, F. Miglior, and B.A. Mallard. 2006. Genetic parameters of dairy cattle immune response traits. In Proceedings of the 8th World Congress on Genetics Applied to Livestock Production; p. 15–18.

[CIT0011] Hickey, M. C., M. Drennan, and B. Earley. 2003. The effect of abrupt weaning of suckler calves on the plasma concentrations of cortisol, catecholamines, leukocytes, acute-phase proteins and in vitro interferon-gamma production. J. Anim. Sci. 81(11):2847–2855. doi:10.2527/2003.81112847x 14601889

[CIT0012] Hine, B. C., A. M. Bell, D. D. O. Niemeyer, C. J. Duff, N. M. Butcher, S. Dominik, A. B. Ingham, and I. G. Colditz. 2019. Immune competence traits assessed during the stress of weaning are heritable and favorably genetically correlated with temperament traits in Angus cattle. J. Anim. Sci. 97:4053–4065. doi:10.1093/jas/skz260 31581299 PMC6776280

[CIT0013] Hodgins, D. C., and P. E. Shewen. 2012. Vaccination of neonates: problem and issues. Vaccine. 30(9):1541–1559. doi:10.1016/j.vaccine.2011.12.047 22189699

[CIT0014] Kirkpatrick, G., D. L. Step, M. E. Payton, J. B. Richards, L. F. McTague, J. T. Saliki, A. W. Confer, B. J. Cook, S. H. Ingram, and J. C. Wright. 2008. Effect of age at the time of vaccination on antibody titers and feedlot performance in beef calves. J. Am. Vet. Med. Assoc. 233:136–142. doi:10.2460/javma.233.1.136 18593324

[CIT0015] Knowles, T. G., J. E. Edwards, K. J. Bazeley, S. N. Brown, A. Butterworth, and R. D. Warriss. 2000. Changes in the blood biochemical and haematological profile of neonatal calves with age. Vet. Rec. 147:593–598. doi:10.1136/vr.147.21.593 11110479

[CIT0016] Larmer, S. G., and B. A. Mallard. 2017. High immune response sires reduce disease incidence in North American large commercial dairy populations. Cattle Pract. 25:74–81.

[CIT0017] Mallard, B. A., M. Emam, M. Paibomesai, K. Thompson-Crispi, and L. Wagter-Lesperance. 2015. Genetic selection of cattle for improved immunity and health. Jpn. J. Vet. Res. 63:S37–S44. doi:10.14943/jjvr.63.supp.s37 25872325

[CIT0018] Mallard, B. A., and B. N. Wilkie. 2007. Phenotypic, genetic and epigenetic variation of immune response and disease resistance and disease resistance traits of pigs. Adv. Pork Produc. 18:139–146.

[CIT0019] Marcos, A., E. Nova, and A. Montero. 2003. Changes in the immune system are conditioned by nutrition. Eur. J. Clin. Nutr. 57:S66–S69. doi:10.1038/sj.ejcn.1601819 12947457

[CIT0020] Okada, H., G. Kohanbash, and M. T. Lotze. 2010. MicroRNAs in immune regulation – opportunities for cancer immunotherapy. Int. J. Biochem. Cell Biol. 42:1256–1261. doi:10.1016/j.biocel.2010.02.002 20144731 PMC2889234

[CIT0021] Richeson, J. T., and T. R. Falkner. 2020. Bovine respiratory disease: what is the effect of timing? Vet. Clin. Food Anim. 36:473–485. doi:10.1016/j.cvfa.2020.03.013 32451036

[CIT0022] Simon, A. K., G. A. Hollander, and A. Michael. 2015. Evolution of the immune system in humans from infancy to old age. Proc. Biol. Sci. 282(1821):20143085. doi:10.1098/rspb.2014.3085 26702035 PMC4707740

[CIT0023] Stear, M., F. C. Karen, J. Nicholas, B. Mallard, and D. Groth. 2017. Genetic variation in immunity and disease resistance in dairy cows and other livestock. In: Webster, J., editor. Achieving sustainable production of milk. Vol. 3. Cambridge, UK: Burleigh Dodds Science Publishing. p. 1–23.

[CIT0024] Thompson-Crispi, K., H. Atalla, F. Miglior, and B. A. Mallard. 2014a. Bovine mastitis: frontiers in immunogenetics. Front. Immunol. 5:493. doi:10.3389/fimmu.2014.00493 25339959 PMC4188034

[CIT0025] Thompson-Crispi, K. A., F. Miglior, and B. A. Mallard. 2013. Incidence rates of clinical mastitis among Canadian Holsteins classified as high, average, or low immune responders. Clin. Vaccine Immunol. 20:106–112. doi:10.1128/CVI.00494-12 23175290 PMC3535773

[CIT0026] Thompson-Crispi, K., M. Sargolzaei, R. Ventura, M. Abo-Ismail, F. Miglior, F. Schenkel, B. A. Mallard. 2014b. A genome-wide association study of immune response traits in Canadian Holstein cattle. BMC Genomics. 15:559. doi:10.1186/1471-2164-15-559 24996426 PMC4099479

[CIT0027] Thompson-Crispi, K., A. Sewalem, F. Miglior, and B. A. Mallard. 2012. Genetic parameters of adaptive immune response traits in Canadian Holsteins. J. Dairy Sci. 95:401–409. doi:10.3168/jds.2011-4452 22192219

[CIT0028] Tierney, T. J., and M. W. Simpson-Morgan. 1997. The proliferative responses of lymphocytes from foetal calves and adult ­cattle. Vet. Immunol. Immunopathol. 59:1–2. doi:10.1016/s0165-2427(97)00057-3 9437825

[CIT0029] Tirado, M. C., R. Clarke, L. A. Jaykus, A. McQuatters-Gollop, and J. M. Frank. 2010. Climate change and food safety. I. A review. Food Res. Int. 43:1745–1765. doi:10.1016/j.foodres.2010.07.003

[CIT0030] Wagter, L. C., B. A. Mallard, B. N. Wilkie, K. E. Leslie, P. J. Boettcher, and J. C. M. Dekkers. 2000. A quantitative approach to classifying Holstein cows based on antibody responsiveness and its relationship to peripartum mastitis occurrence. J. Dairy Sci. 83:488–498. doi:10.3168/jds.S0022-0302(00)74908-3 10750107

[CIT0031] Wilson, R. A., A. Zolnai, P. Rudas, and L. V. Frenyo. 1996. T-cell subsets in blood and lymphoid tissues obtained from fetal calves, maturing calves, and adult bovine. Vet. Immunol. Immunopathol. 53:49–60. doi:10.1016/0165-2427(95)05543-6 8941968

